# Cell polarisation and the immunological synapse

**DOI:** 10.1016/j.ceb.2012.08.013

**Published:** 2013-02

**Authors:** Karen L Angus, Gillian M Griffiths

**Affiliations:** Cambridge Institute for Medical Research, Addenbrooke's Hospital, Cambridge, CB2 0XY, England, UK

## Abstract

Directed secretion by immune cells requires formation of the immunological synapse at the site of cell-cell contact, concomitant with a dramatic induction of cell polarity. Recent findings provide us with insights into the various steps that are required for these processes: for example, the first identification of a protein at the centrosome that regulates its relocation to the plasma membrane; the use of super-resolution imaging techniques to reveal a residual actin network at the immunological synapse that may permit secretory granule exocytosis; and the drawing of parallels between primary cilia and IS architecture. Here we discuss these and other novel findings that have advanced our understanding of the complex process of immunological synapse formation and subsequent induced cell polarity in immune cells.

**Current Opinion in Cell Biology** 2013, **25**:85–91

This review comes from a themed issue on **Cell architecture**

Edited by **Anna Akhmanova** and **Tim Stearns**

For a complete overview see the Issue and the Editorial

Available online 16th September 2012

0955-0674/$ – see front matter, Crown Copyright © 2012 Published by Elsevier Ltd. All rights reserved.

**http://dx.doi.org/10.1016/j.ceb.2012.08.013**

## Introduction

**Figure 1:**
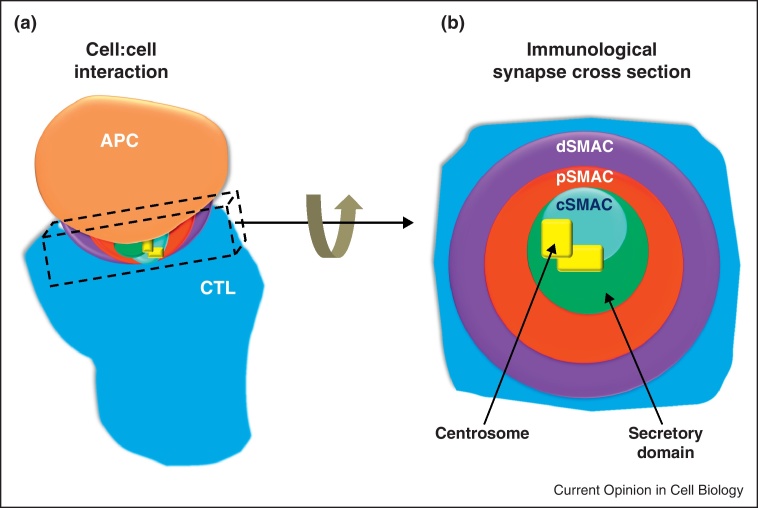
The immunological synapse (IS) in cytotoxic T cells (CTL). The IS forms at the site of cell contact between CTL and APC (a), with a series of supramolecular activation clusters (SMAC) forming as receptors segregate into a characteristic bullseye pattern when viewed *en face* (b) the central SMAC (cSMAC) with clustered T cell receptors (TCRs) involved in target recognition; the peripheral SMAC (pSMAC) with integrins involved in adhesion and the distal SMAC (dSMAC) with excluded phosphatases (CD45) and actin. Polarised secretion from CTL is directed by the centrosome (see Fig. 2) [13], which contacts the cSMAC forming the secretory domain around this point [49].

**Figure 2:**
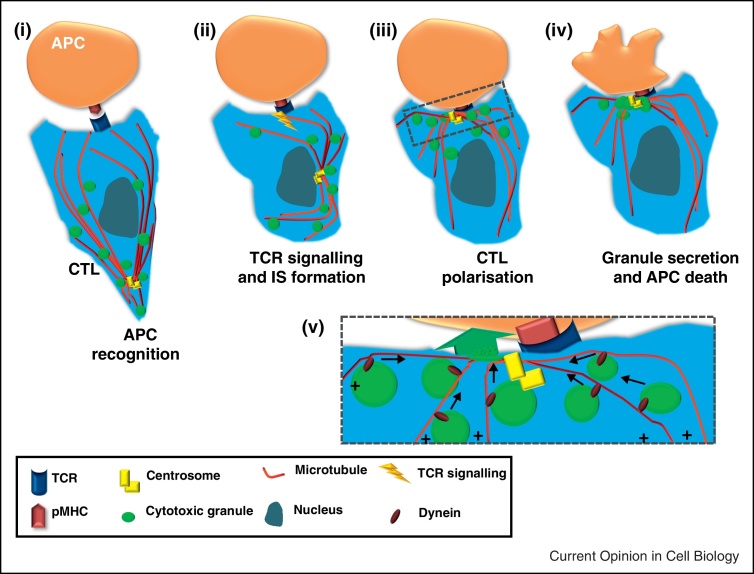
Cell polarity in CTL upon IS formation. (i) Migrating CTL recognise potential target APCs by engagement of peptide-major histocompatibility complex (pMHC) on the APC with cognate T cell receptor (TCR) on the CTL. (ii) Once TCR has engaged with pMHC, signalling within the CTL begins to occur, inducing the formation of the IS and polarisation of the CTL. (iii) The centrosome (which is the microtubule organising centre in CTL) moves from the uropod of the cell where it is found in migrating CTL and repositions itself at the point of TCR signalling. Cytotoxic granules move along microtubules in a minus-end direction towards the polarised centrosome. (iv) Secretion of cytotoxic granules at the secretory domain of the IS induces cell death in the recognised target APC. (v) Zoomed in from box in (iii) showing dynein moving granules in a minus-end direction along microtubules towards the centrosome where they are secreted towards the target cell.

The rearrangement of cell components to form the distinctive immunological synapse (IS), illustrated in Fig. 1, occurs when immune cells polarise in response to recognition of an antigen presenting cell (APC) [1,2]. Receptors involved in APC recognition and intracellular organelles both polarise towards the IS, permitting the transmission of signals and precise secretion towards the APC. IS formation and induction of cell polarity are especially important for cytotoxic T cells (CTL) and natural killer (NK) cells as these events allow the cells to use polarised secretion to destroy APCs, the stages of which are depicted in Fig. 2. Although the IS and cell polarisation in immune cells are tightly linked, we still do not completely understand this interplay, and much of the current research is focused on extending our knowledge in this area. This review will focus on the secretory synapses formed by CTL and NK cells.

## Insights from new techniques

**Figure 3:**
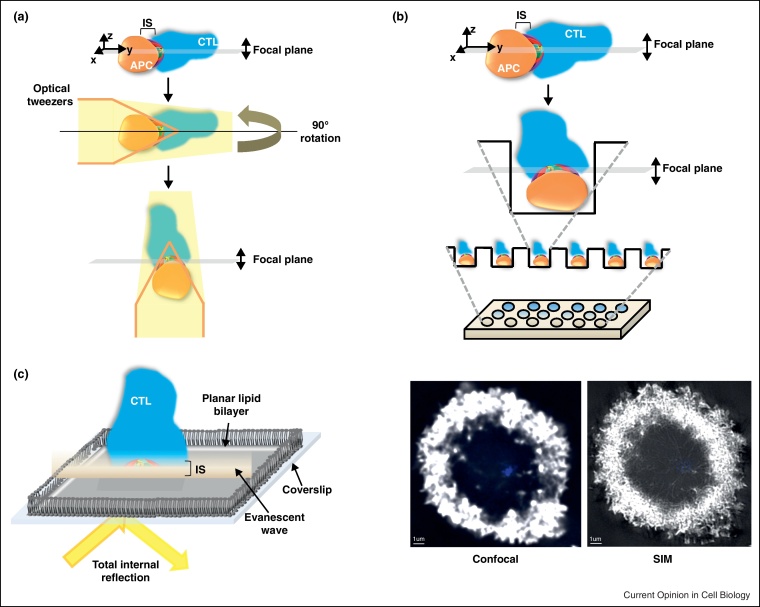
Fluorescence microscopy for study of the IS. (a) An immune cell conjugate, shown here as a CTL and an APC, is trapped using optical tweezers and reorientated. The IS now lies in the focal plane of the confocal microscope allowing high-resolution imaging. The optical tweezers are shown as yellow light surrounding the conjugate with the orange lines representing force generating units required for reorientation. (b) As demonstrated by Biggs [5], a grid can be fabricated with pits into which APCs can be seeded. Upon addition of immune cells to the plate, for example CTL, the size constraints of the pit cause the IS to form in the microscope focal plane, increasing the resolution obtainable. This method allows many conjugates to be prepared in the correct orientation compared to the optical tweezer method due to the number of pits on the array. (c) Immune cells will conjugate to a planar lipid bilayer containing specific activating proteins, as if recognising an APC. In this schematic, a CTL has interacted with a planar lipid bilayer, consequently forming an IS and high-resolution imaging is obtained by total internal reflection microscopy. An evanescent wave, generated upon reflection of light at the coverslip, ascends only 100 nm into the CTL meaning background fluorescence is much reduced and high-resolution of IS components is obtained as only fluorophores in this small illuminated region are activated. (d) A comparison of a widefield deconvoluted confocal image the IS of a T cell activated with poly-l-lysine and anti-CD3 to that obtained using super-resolution structured illumination microscopy (SIM). Images are of the same cell, showing actin in white and the centrosome in blue, achieved by use of Phalloidin-Alexa Fluor 488 (Sigma) and anti-pericentrin (abcam ab4448). SIM reveals more detail at the IS centre as well as at the dSMAC. Scale bars are 2 μm. Imaging was performed using DeltaVision OMX 3D-SIM System V3 (Applied Precision) at the Gurdon Institute, Cambridge, UK. All data capture used an Olympus 100 × 1.4NA oil objective, 405 and 488 nm laser illumination and standard excitation and emission filter sets. 3D-SIM images were sectioned using 125 nm Z-step size. Raw 3-phase images were rendered and reconstructed in 3D by softWoRx 5.0.0 (Applied Precision) software. Images obtained and provided by Nele Dieckmann and Nicola Lawrence, Cambridge, UK.

The dynamic formation of the IS has been uncovered in greater detail recently due to increased use of advanced microscopy techniques. A number of different approaches have been employed to obtain high-resolution images of the cell–cell interface, yielding new insights into the formation of the IS. Those of interest are summarised in Fig. 3 and discussed subsequently.

Optical tweezers can rotate interacting cells so that the interface lies in the higher resolution *xy* plane instead of the *xz* plane (Fig. 3a). This method avoids combining *xy* planes, which generates a low-resolution image in the *z* plane. Its use is nicely demonstrated in an in-depth assessment of interactions of signalling components SLP-76 and LAT, adaptor proteins that are phosphorylated downstream of the TCR thus allowing signal propagation. The study suggests that it is vesicular LAT that is important for signal transduction [3^••^,4]. Using a similar principle, conjugate orientation in micropit arrays also allows positioning of the interface in the horizontal imaging plane [5] (Fig. 3b).

Total internal reflection fluorescence microscopy (TIRFM) on cells adhered to planar lipid bilayers also generates high-resolution images of the IS (Fig. 3c) and has been used extensively to identify the mechanisms controlling the clustering of receptors into the central supramolecular activation complex (cSMAC, Fig. 1b). These assays have revealed receptor microclusters form at the periphery of the IS and migrate inward centripetally, controlled by F-actin flow [6]. Further microcluster TIRFM studies, complemented by super-resolution stimulated emission depletion microscopy (STEDM), revealed dynein-dependent movement along microtubules closer to the IS centre [7^•^], suggesting two distinct phases of movement as shown for B cells [8]. Photoactivatable linkages between T cell receptor (TCR) and peptide-major histocompatibility receptor (pMHC) monomers additionally demonstrated that TCR bound to pMHC are selectively recruited to the cSMAC [9].

Confocal imaging indicates that actin is cleared to the dSMAC of the IS [10], but two simultaneously published papers highlighted the benefits of super-resolution microscopy by revealing a residual actin network across the NK IS where secretion occurs [11^•^,12^•^] (Fig. 3d). This is more noticeable in the higher resolution STEDM images [12^•^] than the 3D-structured illumination microscopy [11^•^]. The authors suggest a role for the residual actin in granule secretion, discussed in ‘Control of granule delivery’.

## Control of centrosome polarisation

A key event in IS induced cell polarisation is movement of the centrosome right up to the membrane at the edge of the cSMAC, initially observed in CTL [13] and more recently in CD4 [14^••^] as well as NK and NKT cells [15^•^] (Figs. 1 and 2). As the centrosome is the microtubule organising centre of CTL, its movement induces reorganisation of the intracellular microtubule cytoskeleton, which is thought to allow polarised secretion of lytic granules at the IS (see ‘Control of granule delivery’ and Fig. 2). Interestingly, although movement of the centrosome and its membrane docking were observed many years ago, the literature is yet to comprehensively explain how this precise and very unusual movement is controlled.

Many studies suggest that the cytoskeleton, along with motor proteins, may permit the centrosome to move to the IS. Dynein and related molecules have long been implicated in this process (Fig. 2v). Huse linked diacylglycerol (DAG) accumulation at the IS with dynein recruitment and centrosome polarisation [16], and more recently depletion of DAG kinase *ζ* revealed its participation in restricting DAG to the IS, although this study did not assess the subsequent effect on centrosome reorientation [17]. Proteins linking the cytoskeleton and membrane also appear to have a role; ezrin localises to the Jurkat IS along with the epithelial cell polarity protein Discs-large homolog 1 (Dlg1) [18]. This work also suggested ezrin regulates Dlg1 because ezrin depletion caused a modest decrease in Dlg1 IS localisation, with depletion of Dlg1 itself having some negative impact on the ability of the centrosome to polarise to the IS. Additionally, expression of key leucine–aspartate domains of paxillin, (a centrosome localised cytoskeletal adaptor protein best known for regulation of focal adhesions [19]) reduced CTL centrosome polarisation by 48% [20], again suggesting that cytoskeletal rearrangements have a role in induced centrosome movement.

Another intriguing candidate is casein kinase I-δ (CKIδ) which, when depleted from Jurkat cells, caused a strong reduction in centrosome polarisation to the IS [21^••^]. What is particularly fascinating about CKIδ is that it is one of the first centrosomal proteins found to influence centrosome positioning towards the IS, although CKIδ regulates microtubule growth and so may well control centrosome positioning in this way. CKIδ interacts with microtubule binding protein EB1 and the p150^glued^ subunit of dynactin, and, since CKIδ localised to the centrosome but was not seen at the IS, these interactions seem more probably to be involved in microtubule anchoring. IQGAP1, which binds microtubules’ plus-ends and links them to the actin cytoskeleton, has been shown to localise with actin in the dSMAC of the IS (Fig. 1) [13]. Interestingly, IQGAP1 knockdown studies in an NK cell line showed decreased centrosome polarisation towards the IS [22]. Furthermore, in mouse CTL devoid of stathmin, a microtubule binding protein, target killing was decreased by 25%, with a similar decrease in centrosome polarisation [23].

Of interest is the fact that stathmin knockout mice also show impaired protein kinase C-θ localisation at the IS [23] and that recently this cSMAC localising kinase was implicated as a major player in centrosome polarisation to the membrane [24]. Such links propose a complicated relationship between proteins segregating at the IS and other cellular components which is proving difficult to dissect. Further studies focussing on the control of centrosome positioning by cSMAC accumulated TCR signalling components (Fig. 1) have only become possible with the development of inducible knock-out models, since many of these proteins are required for T cell development. TCR signalling is drastically reduced in CTL lacking the cSMAC kinase Lck, with residual ERK and calcium signals being sufficient for the centrosome to polarise around the nucleus towards the IS, but the centrosome unable to actually dock at the plasma membrane [25]. Subsequently, secretion is prevented, demonstrating clearly that centrosome docking is essential for granule delivery to the IS and that TCR signalling has a role in this process.

One curious aspect of centrosome polarisation is that CTL killing is, perhaps surprisingly, unimpaired when centrosome movement is restricted by tethering the centrosome to the nuclear membrane. Under these conditions the centrosome is still able to polarise to the IS and CTL kill their targets as efficiently as control CTL [26].

Collectively, these studies support a role for various aspects of cytoskeletal control in promoting centrosome polarisation and docking, as well as TCR signalling, but the mechanism of centrosome movement still necessitates a clearer explanation.

## Control of granule delivery

As stated in ‘Control of centrosome polarisation’, CTL, CD4, NK and NKT cells employ the centrosome for organisation of microtubules’ minus-ends at the point of signalling on the plasma membrane (Fig. 2) [13,14^••^,15^•^], suggesting that these cell types use the same mechanism for polarised secretion at the IS. The minus-end directed movement of cytolytic granules allows precise delivery to the IS [13] and implies a role for motor proteins. Rab7 is required for cytolytic granules to move towards the CTL centrosome via an indirect interaction with the minus-end directed motor dynein [27] and, similarly, NK cell granule convergence on the centrosome also depends on dynein ([28], Fig. 2v). Recent papers have suggested modifications of this scheme. In a report in CTL, knockdown of the plus-end directed motor protein kinesin-1 provided a modest reduction in degranulation, used to support the idea that plus-end movement along short microtubules might mediate the final delivery of granules to the IS [29]. However, since kinesin-1 plays a role in centrosome stability and positioning [30,31] this provides an alternative explanation.

As discussed in ‘Insights from new techniques’, super-resolution imaging revealed a fine actin network remaining across the IS during secretion [11^•^,12^•^]. These results from NK cells, that include a striking EM image of a granule surrounded by the actin network, led to the proposal that the synapse interface is more like a colander with many small actin holes where the centrosome delivers cytolytic granules [12^•^]. This is interesting because actin and non-muscle myosinIIA are thought to be involved in the final steps of cytolytic granule exocytosis [32], including some lysosome related organelles [33]. Furthermore, NK cytolytic granules appear to be surrounded by non-muscle myosinIIA [34], potentially paralleling the detailed picture of exocrine secretion in pancreatic cells in which myosinIIA and IIB facilitate collapse of the secretory granule [32]. In support of this concept are findings from Eric Long's lab in which the granules fuse but do not diffuse away from the membrane [35].

Secretion is not the only role for myosinIIA. A series of recent studies support its role in IS actin reorganisation by taking advantage of small novel probes for polymerised actin, LifeAct and/or Tractin, in combination with the specific myosinIIA inhibitor blebbistatin [36^•^,37,38^•^]. All of these studies reveal myosinIIA is important for centripetal movement of TCR microclusters within the IS by inhibiting actin dynamics and consequently affecting downstream signalling [38^•^].

The final steps of granule secretion still remain something of a mystery, with a number of different SNARE proteins implicated, but their precise sites of action remaining unresolved. This stems, partly, from the fact that the entire secretory pathway contributes to the final readout of secretion. CTL or NK cells deficient in VAMPs4, 7 or 8, Vti1b, or syntaxin7 reveal modest or transient reductions in granule release, suggesting they are involved somewhere in the secretory pathway [39–41]. Even proteins identified from genetic diseases, such as syntaxin11 or Munc18-2 where mutations cause loss of CTL exocytosis *in vivo*, remain difficult to understand since culture with interleukin-2 corrects secretory defects [42,43].

## The immunological synapse as a frustrated cilium

Centrosome docking at the plasma membrane is an unusual event, seen only during cilia formation and cytokinesis (see [44]). This similarity between IS and cilia, first noted by the centrosome docking at the synapse [13], is intriguing because, historically, lymphocytes are thought to be one of a very small number of cell types that do not form primary cilia [45,46]. A number of observations support these parallels including the polarisation of secretory and endocytic organelles to the point of centrosome docking in both cilia and the IS [47], the expression of intraflagellar proteins in lymphocytes [48] and the cilia-like protrusions that form during centrosome docking at the IS [45]. These observations support the idea that centrosome docking leads to the specialisation of an area of plasma membrane for endocytosis and exocytosis, which is critical for both cilia and IS function.

## Conclusions

The IS forms an area of membrane specialisation for polarised secretion from immune cells. This involves reorganisation of not only cell surface receptors, but also actin and microtubule cytoskeletons leading to focused signalling and secretion. In the past couple of years a great deal has been learnt about the mechanisms that control synapse formation. However, further work elucidating a clear pathway that regulates centrosome movement within immune cells is still required. Clarifying the roles of actin and myosins at the IS, as well as proteins required for exocytosis, is crucial in order to resolve current discrepancies in the field. Together these approaches should answer key questions regarding spatial and temporal regulation of IS formation and cell polarisation.

## References and recommended reading

Papers of particular interest, published within the period of review, have been highlighted as:

• of special interest
•• of outstanding interest
